# The underlying mechanisms of DNA methylation in high salt memory in hypertensive vascular disease

**DOI:** 10.1038/s41598-024-51279-1

**Published:** 2024-01-09

**Authors:** Nannan Liu, Yixiao Chen, Yuhan Wang, Sha Wu, Jie Wang, Luming Qi, Tingting Deng, Lina Xia

**Affiliations:** 1 College of Health and Rehabilitation, Chengdu University of Traditional Chinese Medicine, Chengdu, Sichuan China; 2 Child Mental Health Research Center, Nanjing Brain Hospital Affiliated of Nanjing Medical University, Nanjing, Jiangsu China; 3 College of Nursing, Chengdu University of Traditional Chinese Medicine, Chengdu, Sichuan China

**Keywords:** Genetics, Epigenetics

## Abstract

This study demonstrates the effect and DNA methylation-related mechanisms of a high-salt diet and salt memory-induced hypertension and vasculopathy. Thirty Sprague Dawley rats were randomly divided into a control (CON) group (n = 6) and a modeling group (n = 24). A 12% NaCl solution (1 mL/100 g) was intragastrically administered for 60 consecutive days for modeling. An increase in blood pressure up to 140 mmHg was considered successful modeling. Twelve of fifteen successfully modeled rats were randomly selected and divided into a High Salt Diet (HSD) group and a High Salt Memory (HSM) group (n = 6). Rats in HSD group were intragastrically administered a 12% NaCl solution, while rats in HSM group were administered a 3% NaCl solution twice a day for 30 days. At the end of the intervention, blood pressure and the serum levels of ET-1, NO, TNF-α and IL-1β were measured. RRBS-heavy sulfite sequencing technology was selected for DNA methylation analysis. The systolic blood pressure of rats in the HSD group and HSM group was significantly higher than that in the CON group. Compared with those in the CON group, the serum levels of ET-1 in the HSM group and the serum levels of NO in the HSD group and HSM group were significantly increased. The methylation level of the CON group was lower than that of the HSD group and the HSM group, and there was no significant difference between the HSD group and the HSM group. The methylation level of Myoz3 was downregulated in the HSD group and HSM group. The methylation level of Fgd3 were upregulated in HSD group and downregulated in the HSM group. The methylation levels of AC095693.1, Adamts3, PDGFA and PDGFRα were downregulated in the HSD group and upregulated in the HSM group. According to the GO database, the differentially methylated genes were significantly enriched in the coordination of cell function, genetic development, and RNA transcription. There were three main metabolic pathways that were enriched in the differentially expressed genes between the groups: the PI3K-Akt signaling pathway, MAPK signaling pathway, and Hippo signaling pathway. Excessive salt intake may cause hypertension and vascular damage, and this damage may continue after the reduction of salt intake. Therefore, salt memory phenomenon exists, and this memory effect may be correlated with the levels of DNA methylation.

## Introduction

High salt intake is one of the leading dietary risk factors for chronic noncommunicable diseases^1^. Although the World Health Organization (WHO) recommends an amount of dietary salt intake of < 5 g/day, most populations worldwide still consume > 10 g daily^2^. Excessive salt intake promotes cognitive impairment^3,4^, increases the risk of stroke^5^ and is a main risk factor for developmental hypertension, which leads to cardiovascular disease^6–8^. The vessels of patients with arterial hypertension show varying degrees of vascular dysfunction. Subclinical organ damage, such as vascular dysfunction, which is an intermediate stage in the continuum of vascular disease and a determinant of total cardiovascular risk, has great importance in patients with arterial hypertension. The vascular endothelium plays a fundamental role in modulating vascular tone and structure. Disorder of endothelial function in hypertension is likely to be caused in part by genetic factors and is not just a consequence of elevated blood pressure^9,10^.

High salt intake leads to an imbalance of ion homeostasis, activation of the sympathetic nerve and renin–angiotensin–aldosterone system (RAAS), insulin resistance, and other pathophysiological changes, resulting in damage to multiple organs, such as the heart, brain, kidney, and blood vessels^11,12^. Excessive salt in tissue promotes the enrichment of proinflammatory immune cells in an osmotic pressure-dependent manner^13,14^. A study showed that transient high salt intake during early phases in the development of hypertension induces a sustained elevation of BP in hypertensive model rats. Therefore, the salt memory phenomenon exists^15^. Jax^16^ proposed that microvascular changes mediated by hyperglycemia could play an important role in the development of metabolic memory observed in the Diabetes Control and Complications Trials in diabetes mellitus. Oguchi^15^ speculated that the change in renal microvasculature through an increase in blood pressure (BP) at a certain important period plays a major role in the occurrence of salt memory. Long-term high salt intake will lead to the excessive synthesis and release of eosinophils and inhibit sodium pumps, thus increasing the intracellular Na^+^ content. Moreover, increased sodium and calcium exchange will lead to calcium overload, resulting in contraction of intravascular smooth muscle, increased resistance of perivascular smooth muscle, and increased blood pressure^17^.

It is well known that essential hypertension is closely related to genetic factors. Furthermore, a large number of studies have shown that essential hypertension and environmental factors are also closely connected. Science plays a crucial role as a bridge in this pathological process, closely linking genetic factors and environmental factors, which provides a new perspective for our study of essential hypertension. Deoxyribonucleic Acid (DNA) methylation is the earliest epigenetic regulatory mechanism recognized by humans and plays an important role in environmental adaptation, embryonic development, cell differentiation, and disease occurrence and development. It plays an important role in the occurrence and development of essential hypertension^18^. The gene promoter region undergoes a certain degree of methylation or demethylation under certain circumstances, and during this process, it causes changes in the expression levels of some related enzymes and receptors. Changes in those related to blood pressure regulation can increase blood pressure.

DNA methylation can be involved in the immune response and inflammatory response through interactions with immune pathways. Studies have shown that the proinflammatory factor IL-1β can affect the DNA methylation pattern by altering the expression of DNA Methyltransferase (DNMT). This leads to changes in inflammatory factor expression and abnormal functional activities of cells in the body, thus affecting the activation of intestinal epithelial cells^19^. Other studies have found that proinflammatory factors can increase the expression levels of DNMT1, and its expression level is closely related to the degree of DNA methylation in T lymphocytes. DNA methylation can regulate the activation, differentiation and migration of T lymphocytes, and T lymphocyte activation can lead to demethylation of the anti-inflammatory factor interleukin-2 promoter region. Thus, the expression level of the interleukin-2 gene is increased^20^. In vivo and in vitro data suggest that DNMT1 can activate macrophages and promote the expression of the proinflammatory cytokines IL-6 and TNF-α by inhibiting KLF-4 expression^21^. The interaction between DNA methylation and the inflammatory response jointly promotes the occurrence of hypertensive vasculopathy by influencing the regulation of gene expression, DNA methylation, response to antigen stimulation and selective splicing. This study was designed to demonstrate the effect and epigenetic mechanisms of high salt memory-induced hypertension and vasculopathy by observing the serum levels of endothelin-1 (ET-1), Nitric Oxide (NO), Tumor Necrosis Factor-α (TNF-α), and Interleukin-1β (IL-1β) and DNA methylation in heart tissue.

## Materials and methods

### Animals

Thirty healthy Specific Pathogen Free (SPF) Sprague-Dawley (SD) rats with an age of 6–8 weeks and a body weight of 200 ± 20 g were selected. Since the estrogen level in female rats will affect the regulation of blood pressure, all experimental rats were male. The rats were purchased from the Sichuan Academy of Chinese Medicine Sciences (license number: SYXK (Sichuan) 2018-100). Animal care was carried out in accordance with the Instruction for Ethical Treatment of Animals issued by the Ministry of Science and Technology, China, 2006. We tried to minimize the number and suffering of laboratory animals. All procedures and animal experiments were approved by the Animal Care and Use Committee of Chengdu University of Chinese Medicine (Protocol number: 2018-21). All methods were performed in accordance with the relevant guidelines and regulations. The reporting in the manuscript follows the recommendations in the ARRIVE guidelines.

### Pharmaceutical preparation

NaCl solution: Pure sodium chloride and distilled water were used to prepared a 12% NaCl solution at a ratio of 0.12:1 (the concentration was obtained according to the animal model of blood stasis syndrome constructed by the research group under the guidance of the theory of "salt wins blood" in traditional Chinese medicine^24^).

### Grouping and modeling

After adaptive feeding for 1 week, 30 rats were randomly divided into two groups, the control (CON) group (6 rats) and the model group (24 rats), with free access to an ordinary diet (containing 0.3% salt) and normal drinking water.

For the CON group, intragastric administration of 0.9% NaCl solution (1 mL/100 g) was carried out once every morning for 60 consecutive days.

For the model group, intragastric administration of 12% NaCl solution (1 mL/100 g) was carried out once every morning for 60 consecutive days.

The body weight was measured once a week. After 60 days, blood pressure was measured, and a significant increase in blood pressure (P < 0.01) up to 140 mmHg was considered to indicate successful modeling. Fifteen rats were considered to have achieved successful modeling. Twelve rats were randomly selected and divided into two groups, the High salt diet (HSD) group and the High salt memory (HSM) group, with six rats each.

### Administration

On the second day of successful modeling, rats in the HSD group were intragastrically administered 12% NaCl solution (1 mL/100 g), while rats in the HSM group were intragastrically administered 3% NaCl solution (1 mL/100 g). The CON group was given 0.9% NaCl (1 mL/100 g) solution by gavage. The intragastric administration was carried out twice a day with at least 2 h intervals for 30 days.

At the end of the intervention, blood pressure was measured, and the animals were anesthetized after fasting for 12 h by isoflurane inhalation. Under anesthesia, the abdominal aorta and heart tissue were immediately removed, and 5 mL of whole blood was collected. Blood samples were placed in EP tubes sterilized by a high temperature autoclave and left for 30 min. The operating temperature of the centrifuge was 4 °C. Serum was extracted with a pipette gun and stored at − 80 °C.

### SBP measuring

On Days 0, 15, 30, 45, 60, 75 and 90 of the experiment, the tail blood pressure of each rat was measured by a noninvasive blood pressure meter three times from 9:00 to 12:00 a.m., and the average value was recorded. A small amount of water was provided during this period to prevent high-temperature discomfort.

### ELISA

The serum levels of ET-1, NO and the inflammatory factors TNF-α and IL-1β related to vascular endothelial function were determined by double antibody sandwich enzyme-linked immunosorbent assay according to the description of the detection kit: Rat NO ELISA Kit NJJC A013-2-1 (Nanjing Jiancheng), Rat ET-1 ELISA Kit E-EL-R1458c (Elabscience), Rat IL-1β ELISA kit ER008-96 (ExCell), Rat TNF-α ELISA kit ER006-96 (ExCell). Standard substances and serum samples were tested. They were placed on a micro-oscillator (frequency 300 RPM) and incubated at room temperature (20–25 °C) for 120 min. The plate was washed 5 times. Then, 100 μL of enzyme conjugate working solution was added to each well, placed on a micro-oscillator (frequency 300 RPM), and incubated at room temperature (20–25 °C) for 60 min. The plate was washed again 5 times. Next, 100 μL of chromogenic substrate was added to each well and incubated for 15 min at room temperature (20–25 °C) in the dark. Then, 100 μL of stop solution was added to each well and mixed. The OD450 value was measured within 10 min.

### Extraction, quality inspection and on-board sequencing of sample DNA

First, 10–50 mg of heart tissue was minced. Then, 20 mL of proteinase K and 300 mL of tissue digestion solution were added, and an electric grinder was used to completely grind the heart tissue. The tissue was digested at 65 °C for 30 min. After sample digestion, the sample was centrifuged at 12,000 rpm for 1 min to remove residual impurities. Then, 300 mL of lysis solution GHL and 300 mL of isopropanol were added and mixed by shaking. Fifteen milliliters of the suspension was added to an EP tube. The mixture was shaken for 1 min to mix well and allowed to stand for 2 min. and this process was repeated 3 times. The EP tube was placed on the magnetic stand for 30 s. After the DNA of the sample was completely adsorbed by the magnetic beads, the liquid was gently aspirated. The purity of DNA samples was tested (OD260/OD280 ≥ 1.7, OD260/OD230 ~ 2.0). Accurate quantification of the concentration of DNA samples was performed (ensure that the total DNA concentration is ≥ 10 ng/µL). Agarose gel electrophoresis (1%) was used to test the integrity of the DNA. DNA samples were obtained, and the DNA was segmented by the MspI digestion method. Then, the DNA fragments were purified, the end was repaired, A was added at the 3′ end, and methylated linkers were added. Agarose gel electrophoresis was used to select DNA fragments of 230–380 bp, which contained linkers of 100 bp. After salt (bisulfite) treatment, PCR amplification was performed to form a sequencing library. Reads with adapters were filtered out. After the construction of the sequencing library was completed, the concentration of the library was detected. Then, the effective concentration of the library was accurately and quantitatively measured to ensure that the library construction was effective, and the library data were entered into the computer for sequencing.

### Detection of 5-methylcytosine (5mC) in the sample and reference genomes

Statistics were performed on the reads data files that were filtered out. When the statistics were completed, the only valid reads of the reference genome were located by the program BISMARK. Then, the proportion of only the valid reads that were mapped was counted (%). Additionally, the nonmethylation conversion rate (%) was calculated. For the reference genome and sequencing read preprocessing, C- > T conversion was performed on the read of the reference genome, and G- > A conversion was performed on the sequenced read to obtain two versions of the reference genome and two versions of the sequencing read. The converted reads (C- > T and G- > A) were aligned pairwise with the converted reference genome genotypes, and the best and unique alignment results were screened for subsequent analysis. BISMARK software was used for 5mC detection. If there was a C site aligned with Ueyuan genome C, the site was considered to be methylated C, and different types of methylated C were represented by different capital letters. For each site C, the coverage of methylated C and unmethylated C was calculated.

### Detection of Different Methylation Region (DMR) and DMR-related gene annotation

MOABS software was used to screen the DMR regions to detect the presence of DMR between samples. The coverage depth was not less than 4X. There were at least 3 differentially methylated sites, and the minimum difference in methylation level was 0.2. Fisher's exact test was used, and P < 0.05 was considered to indicate statistical significance. According to the position of the DMR on the genome and the annotation information of the genome, the 3000 bp upstream of the gene was taken as the promoter region, and the DMR was annotated. The DMR-associated genes were aligned with the GO and KEGG functional databases to obtain the annotations of these genes to analyze gene functions.

### Statistical analysis

SPSS 25.0 software was used for statistical analysis of the data obtained from the experiment. The Shapiro‒Wilk test was used for the normality test. Variables in accordance with a normal distribution are expressed as the mean ± standard deviation (¯X ± S), and categorical variables are expressed as frequencies. For the data that were different between groups that met normality and homogeneity of variance, one-way analysis of variance was used for data analysis; otherwise, the rank sum test was used for data analysis. Pearson correlation analysis was used to analyze the correlation between the two sets of data. Under normal circumstances, P < 0.05 is considered to indicate statistical significance.

### Ethics approval and consent to participate

All procedures and animal experiments were approved by the Animal Care and Use Committee of Chengdu University of Chinese Medicine (ethical code: 2018-21; 8/3/2021).

## Results

### Salt memory causes the blood pressure of rats to continue to rise

**Figure 1:**
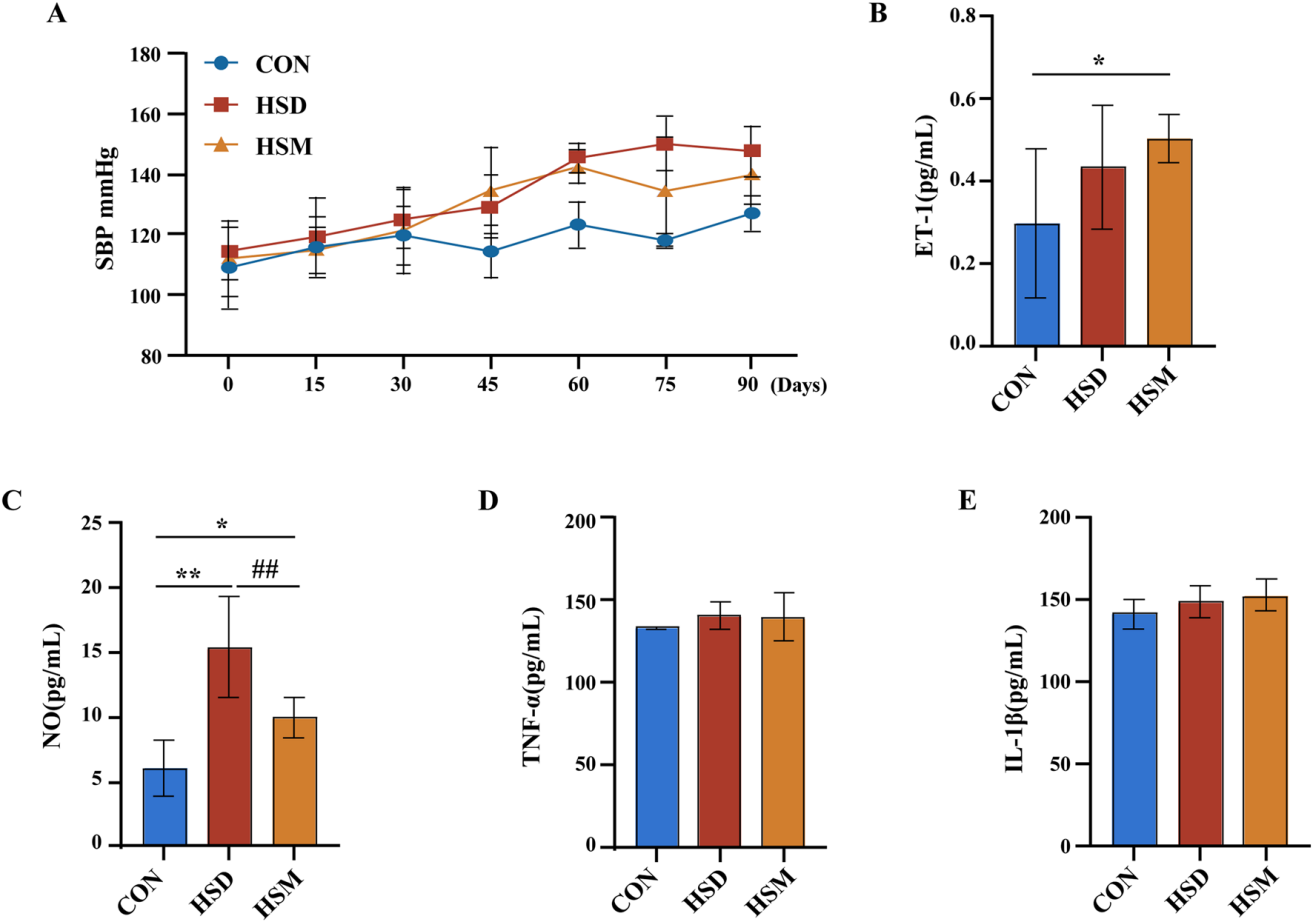
(**A**) Systolic blood pressure (SBP) of rats over 90 days. (**B**) Serum levels of ET-1. (**C**) Serum levels of NO. (**D**) Serum levels of TNF-α; (**E**) Serum levels of IL-1β. *P < 0.05(compared with CON group); **P < 0.01(compared with CON group); ^#^P < 0.05(compared with HSD group).

The systolic blood pressure (SBP) of rats in the HSD group continued to rise after intragastric administration of 12% NaCl. On the 60th day of modeling, the SBP levels of rats in both the HSD group and HSM group were significantly higher than that in the CON group (P < 0.01), and these levels exceeded 140 mmHg. Thus, high salt can cause high blood pressure in rats. There was no significant difference between the HSM group and the HSD group after decreased salt intake for the HSM group, implying that salt memory can lead to a continuous influence on blood pressure (Fig. 1A).

### Salt memory may cause vascular endothelial injury

ET-1 and NO are vasomotor factors secreted by vascular endothelial cells (VECs), which are important factors that reduce the stability of the vascular environment and cause hypertension. Compared with that in the CON group, the serum level of ET-1 in the HSD group was higher (P = 0.147), and the serum level of ET-1 in the HSM group was significantly higher (P = 0.033). Compared with that of the HSD group, the serum level of ET-1 of the HSM group was slightly higher, but this difference was not significant (P = 0.462) (Fig. 1B). Compared with that in the CON group, the serum NO level in the HSD group and HSM group was significantly higher (P = 0.000; P = 0.027). Compared with that in the HSD group, the serum NO level in the HSM group was lower (P = 0.003) (Fig. 1C).

TNF-α is a proinflammatory factor that appears at the very early stage of the inflammatory response. Il-1β is an important mediator that triggers the inflammatory response and promotes inflammation. Compared with that of the CON group, the serum TNF-α level of the HSD group showed an increasing trend (P = 0.085). There was no significant difference between the HSD group and the HSM group (P = 0.777) (Fig. 1D). Compared with that in the CON group, the serum level of IL-1β in the HSD group was slightly higher, but the difference was not significant (P = 0.399). Compared with that in the HSD group, the serum IL-1β level in the HSM group was not significantly different (P = 0.666) (Fig. 1E).

### DNA methylation sequencing analysis of rat heart tissue

There were no high-error bases within the range of 150 nt sequencing length, and the identification error rate of a single base was less than 0.02%. A base mass value ≥ 30 in all sequencing samples accounted for more than 89.44% of the total number of bases, which met the requirements of sequencing follow-up processing and analysis. Clean reads were obtained by filtering low-quality data from the original sequences. The evaluation results of sequencing data of each sample are shown in Table 1. Bases with mass value ≥ 20 (Q20) and mass value ≥ 30 (Q30) accounted for 96% and 89% of the total number of bases, respectively, meeting the requirements of subsequent analysis. The percentage of unique reads on the reference genome in the total number of clean reads that can be located was assessed. Compared with the reference genome, 1.66 × 108 double-ended sequencing clean reads were obtained, and a total of 1.04 × 108 reads were matched to the unique position of the rat reference genome. The average efficiency of comparison between the 9 samples and the reference genome was approximately 62.78%. The bisulfite ratio efficiency was approximately 99.47%, meeting the requirements of subsequent analysis, as shown in Table 2.

**Table 1:** Sequencing data of 9 samples.

Sample	Clean reads	Clean base	GC	Q20	Q30
HSD 1	20,076,792	5,646,073,935	27.67	96.76	90.59
HSD 2	17,380,699	4,868,099,691	27.26	96.66	90.35
HSD 3	17,410,799	4,901,213,380	27.74	96.69	90.45
HSM 1	17,521,479	4,893,669,140	27.12	96.93	91.00
HSM 2	17,184,310	4,807,375,863	27.28	96.45	89.89
HSM 3	15,799,189	4,428,953,446	27.41	96.57	90.16
CON 1	18,095,520	5,081,246,190	27.71	96.84	90.81
CON 2	17,537,412	4,871,904,223	26.69	96.28	89.77
CON 3	22,498,587	6,200,522,875	26.79	96.12	89.44

Clean_bases: the number of bases after filtration; GC: the proportion of base types G and C in the total base of the sample.

**Table 2:** Comparison of the reference genome and 9 samples.

Sample	Clean reads	Unique mapped reads	Mapping rate (%)	Bisulfite conversion rate (%)
HSD 1	20,076,792	13,237,904	65.94	99.51
HSD 2	17,380,699	11,558,184	66.5	99.53
HSD 3	17,410,799	11,897,518	68.33	99.51
HSM 1	17,521,479	10,310,948	58.85	99.51
HSM 2	17,184,310	10,990,275	63.96	99.56
HSM 3	15,799,189	10,203,043	64.58	99.53
CON 1	18,095,520	11,644,898	64.35	99.49
CON 2	17,537,412	9,741,862	55.55	99.09
CON 3	22,498,587	12,897,653	57.33	99.59

Unique mapped: number of clean reads that are uniquely mapped to the reference genome. Mapping rate: the percentage of the number of clean reads located to the reference genome in all the clean reads. Conversion rate: conversion rate of heavy sulfite.

**Figure 2:**
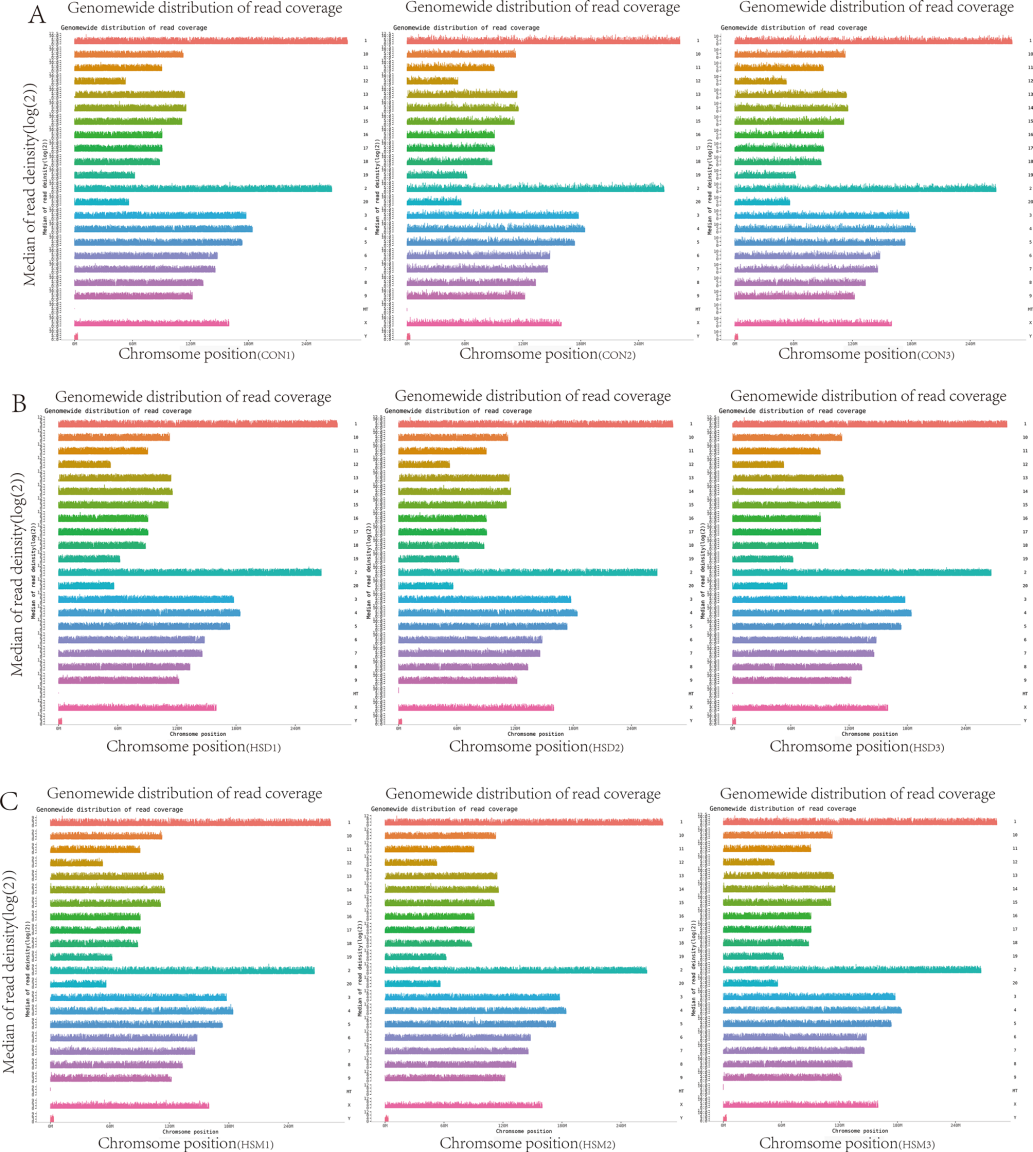
Sample chromosome coverage depth distribution map. (**A**) CON group, (**B**) HSD group, (**C**) HSM group. The x-coordinate is the chromosome position, and the y-coordinate is the pair value of the coverage depth of the corresponding position on the chromosome.

The map was drawn according to the coverage depth of all cytosine sites on the chromosome. If the coverage depth was evenly distributed on each chromosome, the randomness of sequencing was considered to be good. Figure 2 shows the distribution of chromosome coverage depth of 9 samples. DMR is mainly distributed on chromosomes 1–12, and very few are distributed on the Y chromosome and mitochondrial (MT) chromosome.

### 5mC detection and annotation

**Figure 3:**
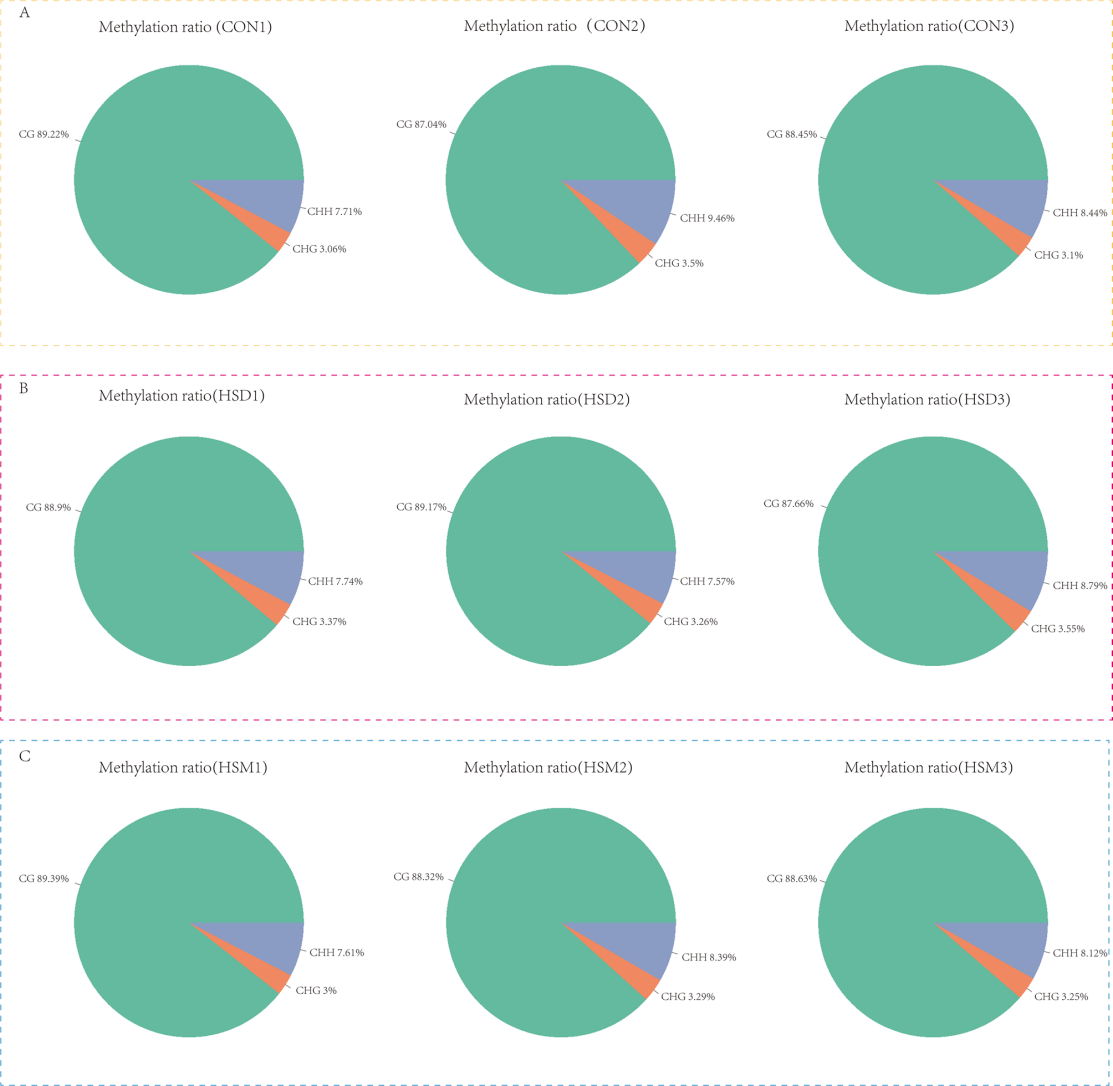
Map of different types of 5mC in the sample genome. (**A**) CON group, (**B**) HSD group, (**C**) HSM group.

**Figure 4:**
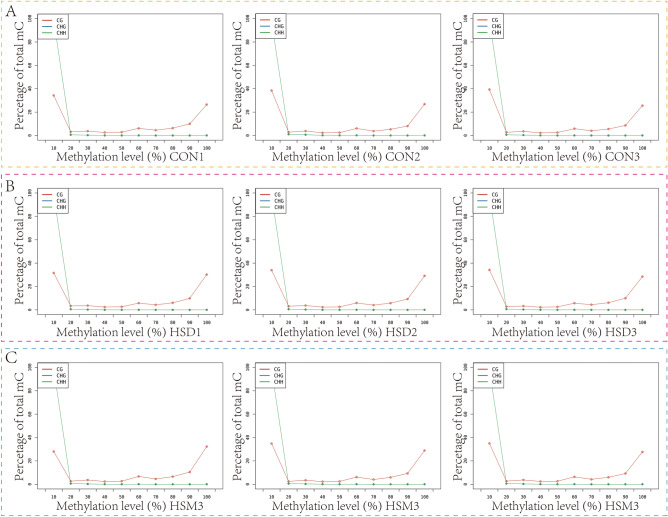
Single base methylation profile of the sample. (**A**) CON group, (**B**) HSD group, (**C**) HSM group. The horizontal axis shows the methylation level from low to high, with each 10% as a region, and the vertical axis shows the percentage of all methylated C bases at a given methylation level.

5mC detection and annotation were carried out based on the binomial distribution test principle, and 5mC detection was performed for each C site. According to the link where the methylated Cytosine is located, there are three categories: CpG, CHG, CHH. Where p represents the phosphodiester bond, and CpG refers to the downstream of the methylated Cytosine is a G base. H represents bases other than G, that is, any one of A, C, T. The proportions of different types of methylation sites on the reference genome are shown in Table 3. CG mC accounted for 87.04–89.39%. CHH mC accounted for 7.57–9.46%, and CHG mC accounted for 3.00–3.55%. This indicated that the restriction enzyme MspI was used in Reduced representation bisulfite sequencing (RRBS) with higher digestion efficiency. In this study, two types of mC distributions, mCG and mCHH, were statistically analyzed in 9 samples, as shown in Fig. 3. The results showed that most mCG sites were mainly concentrated in the region with methylation levels ≤ 40%, while most mCHH sites were mainly concentrated in the two regions with methylation levels ≤ 20% and ≥ 90%. The overall CG type C methylation level of 9 samples was detected, as shown in Fig. 4. The overall methylation level of the CON group was lower than that of the HSD group and the HSM group, and there was no significant difference between the HSD group and HSM group.

**Table 3:** Different types of 5mC in the sample genome.

Sample	CG (+)	CHG (+)	CHH (+)	CG (−)	CHG (−)	CHH (−)	CG (%)	CHG (%)	CHH (%)
HSD 1	1,025,385	38,488	89,692	1,022,123	39,051	88,532	88.9	3.37	7.74
HSD 2	883,453	32,279	75,197	878,112	32,024	74,346	89.17	3.26	7.57
HSD 3	936,803	37,700	93,974	930,951	37,903	93,242	87.66	3.55	8.79
HSM 1	1,072,945	36,197	91,212	1,063,867	35,585	90,707	89.39	3.00	7.61
HSM 2	831,986	31,016	78,755	826,420	30,821	78,752	88.32	3.29	8.39
HSM 3	811,554	29,765	74,450	806,244	29,588	73,703	88.63	3.25	8.12
CON 1	1,006,041	34,562	87,521	999,842	34,341	85,833	89.22	3.06	7.71
CON 2	811,808	32,726	88,229	809,280	32,534	87,942	87.04	3.5	9.46
CON 3	966,575	33,499	90,939	959,076	34,020	92,901	88.45	3.1	8.44

CG, CHG, CHH (±): the number of CpG, CHG, CHH methylation sites detected (positive/negative chain); CG, CHG, CHH (%): percentage of methylated C quantity corresponding to CG, CHG, and CHH types.

### Differential methylation region (DMR) analysis

DMR refers to the same region with different methylation levels in multiple samples and is considered a functional region that may be involved in the regulation of genomic transcription levels. The DMR between samples reflects the difference in methylation level, and the DMR in the promoter region can cause a change in the gene expression level. The number of DMRs obtained after pair-based comparative analysis of methylation regions of the three groups of samples is shown in Table 4. The results showed that there were significant differences in methylation levels among the HSD group, HSM group and CON group, but the differences between the HSD group and HSM group were small.

**Table 4:** Genome-wide DMR number.

Groups	DMR number
HSD vs. HSM	4208
CON vs. HSD	4941
CON vs. HSM	5598

**Figure 5:**
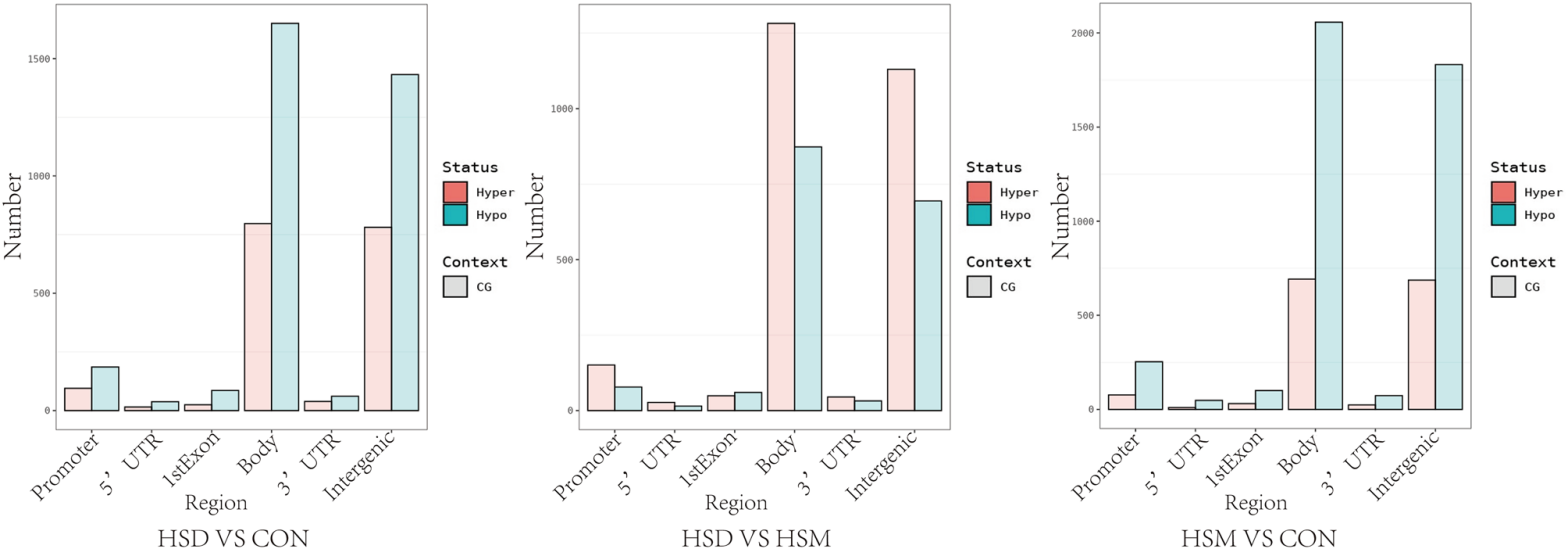
DMR annotation results. The horizontal coordinate is the functional region of the gene, and the vertical coordinate is the corresponding number of DMRs. Different methylation states are represented by different colors.

DNA methylation either weakens or enhances the transcriptional efficiency of the promoter region through methylation or demethylation of multiple CpG sites, thus affecting the gene expression level. As shown in Tables 5, 6 and 7 and Fig. 5, comparative analysis of differential methylation of promoter regions between the HSD group and CON group, the HSD group and HSM group and between the HSM group and CON group showed that methylation levels of promoter regions with 15 genes were changed (P < 0.05). There were 6 genes related to cardiovascular and cerebrovascular diseases, which were Fgd3, Myoz3, AC095693.1, Adamts3, PDGFA and PDGFRα. Compared with the CON group, the methylation level of Myoz3 was downregulated in the HSD group and HSM group, the methylation levels of Fgd3 were upregulated in HSD group and downregulated in the HSM group, the methylation levels of AC095693.1, Adamts3, PDGFA and PDGFRα were downregulated in the HSD group and upregulated in the HSM group. Compared with the HSM group, the methylation levels of Fgd3 were upregulated in the HSD group, the methylation level of Myoz3 was downregulated in the HSD group, the methylation levels of AC095693.1, Adamts3, PDGFA and PDGFRα were downregulated in the HSD group.

**Table 5:** Differentially methylated genes in the promoter region (HSD group vs. CON group).

Gene	Meth direction	Meth Dif	P value	Annotation
Fgd3	strongHyper	0.268	4.04E − 10	Promoter (≤ 1 kb)
Myoz3	strongHypo	− 0.258	3.59E − 34	Promoter (≤ 1 kb)
AC095693.1	strongHypo	− 0.209	2.57E − 08	Promoter (≤ 1 kb)
Adamts3	strongHypo	− 0.212	1.96E − 05	Promoter (≤ 1 kb)
PDGFRα	strongHypo	− 0.245	0.000192	Promoter (2–3 kb)
PDGFA	strongHypo	− 0.342	3.67E − 06	Promoter (1–2 kb)

Methylation direction indicates methylation level. Annotation as a promoter refers to the region to which genes are annotated.

**Table 6:** Differentially methylated genes in the promoter region (HSD group vs. HSM group).

Gene	Meth direction	Meth Dif	P value	Annotation
Fgd3	strongHyper	0.248	7.58E − 09	Promoter (≤ 1 kb)
Myoz3	strongHypo	− 0.275	2.46E − 48	Promoter (≤ 1 kb)
AC095693.1	strongHypo	− 0.218	3.98E − 08	Promoter (≤ 1 kb)
Adamts3	strongHypo	− 0.217	7.47E − 07	Promoter (≤ 1 kb)
PDGFRα	strongHypo	− 0.264	0.00616	Promoter (2–3 kb)
PDGFA	strongHypo	− 0.354	1.41E − 26	Promoter (1–2 kb)

Methylation direction indicates methylation level. Annotation as a promoter refers to the region to which genes are annotated.

**Table 7:** Differentially methylated genes in the promoter region (HSM group vs. CON group).

Gene	Meth direction	Meth Dif	P value	Annotation
Fgd3	strongHyper	0.262	0.00526	Promoter (≤ 1 kb)
Myoz3	strongHypo	− 0.436	8.04E − 12	Promoter (≤ 1 kb)
AC095693.1	strongHyper	0.659	1.70E − 13	Promoter (≤ 1 kb)
Adamts13	strongHyper	0.38	3.27E − 06	Promoter (≤ 1 kb)
PDGFRα	strongHyper	0.375	3.61E − 07	Promoter (2–3 kb)
PDGFA	strongHyper	0.284	1.12E − 05	Promoter (1–2 kb)

Methylation direction indicates methylation level. Annotation as a promoter refers to the region to which genes are annotated.

### Differential methylation GO functional annotation and enrichment analysis

**Figure 6:**
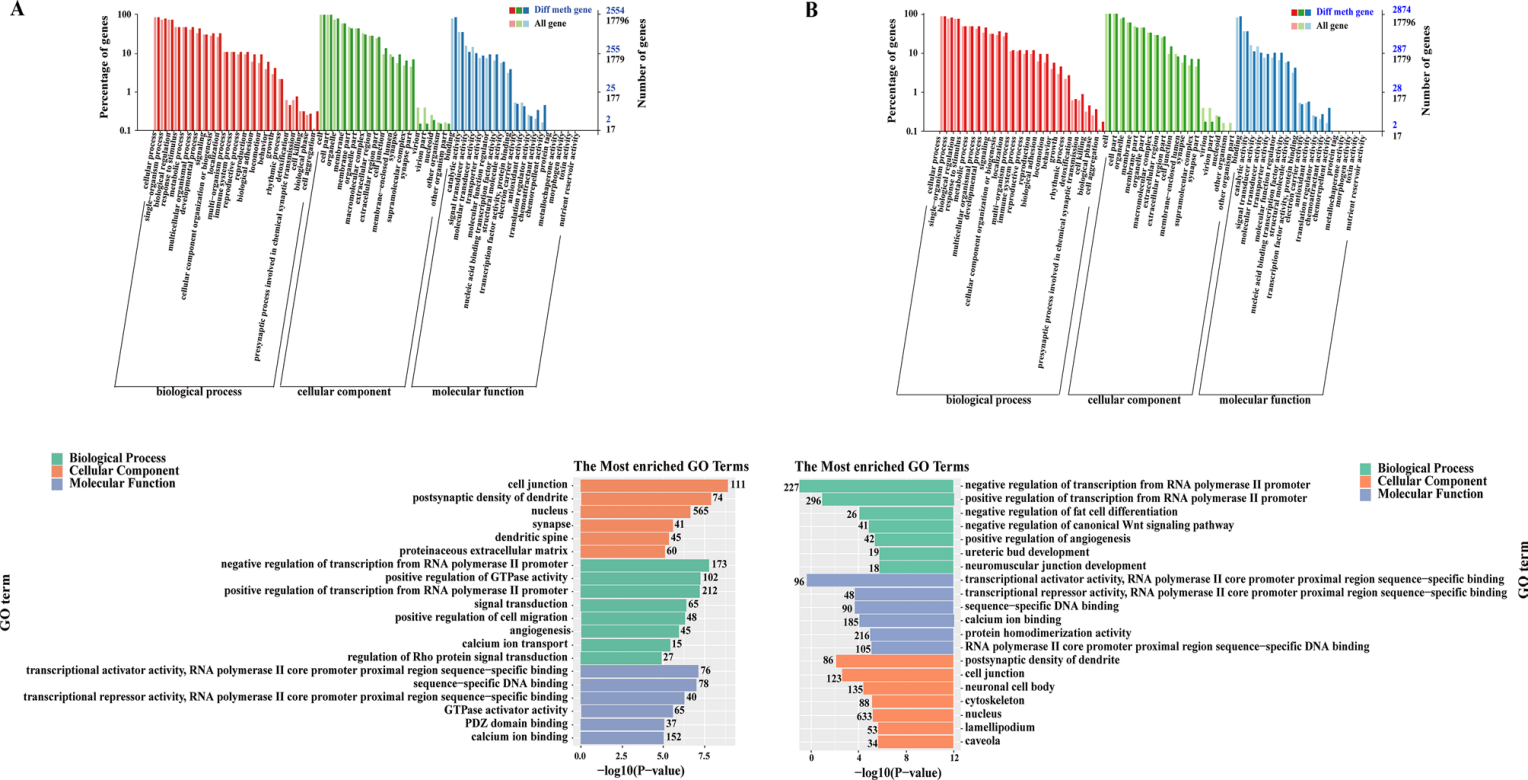
GO functional annotation enrichment map. (**A**) HSD VS CON; (**B**) HSM VS HSD. The red areas are biological processes, the green areas are cellular components, and the blue areas are molecular functions. The right side of the vertical axis is the number of genes, and the left side is the percentage of genes.

GO functional annotation and enrichment analysis were carried out on genes with significant differences in DNA expression levels, and the results are shown in Fig. 6. The differentially expressed genes associated with cardiovascular and cerebrovascular disease were enriched (P < 0.05). The related biological processes included “negative regulation of transcription from RNA polymerase II promoter (GO: 0000122)”, “in utero embryonic development (GO: 0001701)”, “positive regulation of cell migration (GO: 0030335)”. The associated cell component included “cell junction (GO: 0030054)”, “nucleus (GO: 0005634)”, “caveola (GO: 0005901)”. The related molecular functions included “transcriptional activator activity, RNA polymerase II core promoter proximal region sequence-specific binding (GO: 0001077)”, “sequence-specific DNA binding (GO: 0043565)”, “calcium ion binding (GO: 0005509)”, “RNA polymerase II core promoter proximal region sequence-specific DNA binding (GO: 0000978)”. In conclusion, in the GO database, differentially methylated genes were significantly enriched in the coordination of cell function, genetic development, RNA transcription, DNA binding, calcium ion channels and other aspects.

### Differential methylation KEGG pathway enrichment analysis

**Figure 7:**
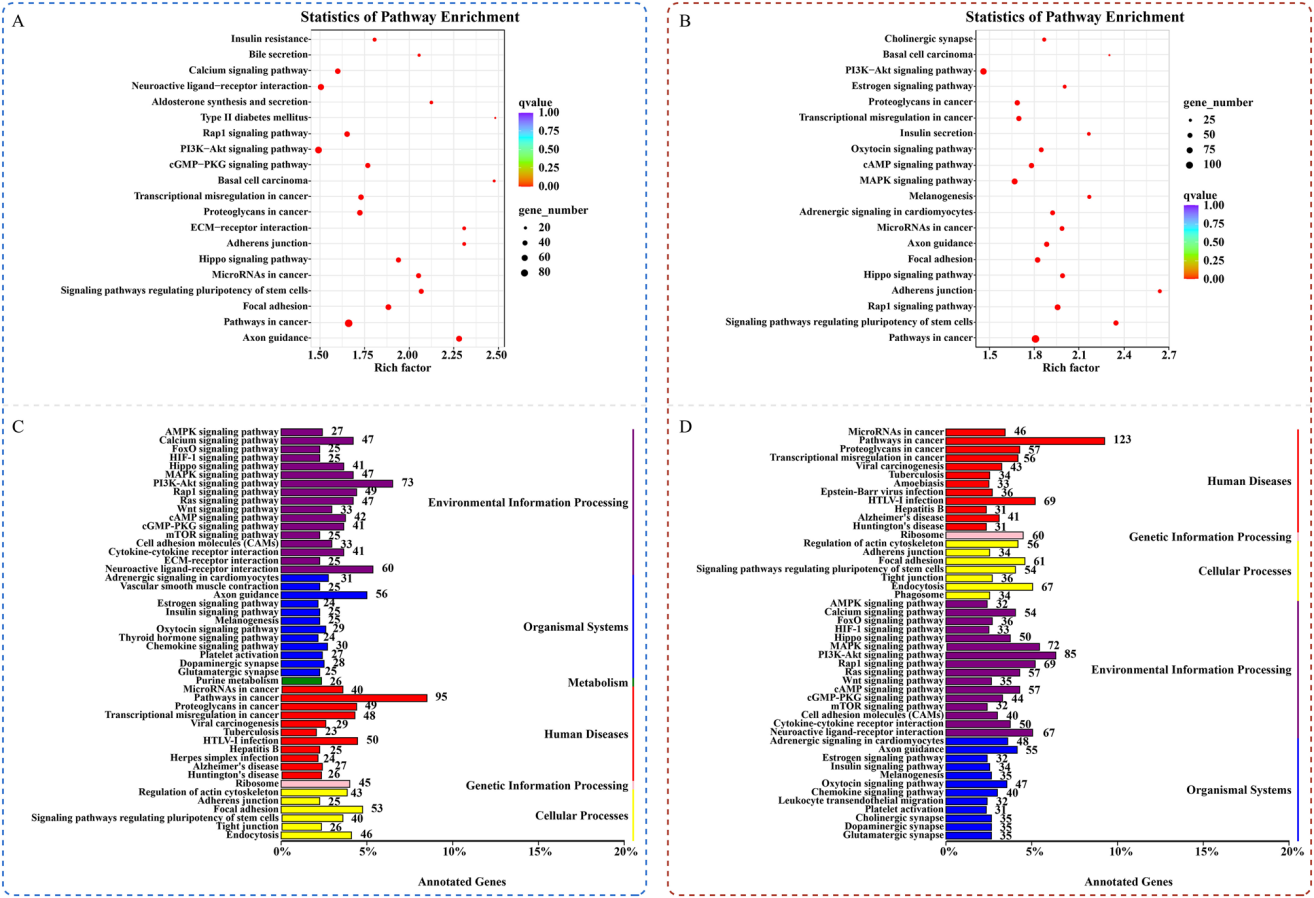
KEGG pathway enrichment map. (**A**, **C**) HSD VS CON, (**B**, **D**) HSM VS HSD. (**A**, **B**) The size of the circle represents the number of genes contained in the class. The larger the circle is, the more significant the enrichment of the class. The different colors of the circle indicate the degree of false-positive enrichment. The smaller the circle is, the lower the false-positive rate.

The KEGG annotation results of DMR-associated genes are classified according to the KEGG pathway type. KEGG pathway enrichment analysis was conducted for genes with significant differences in RNA expression levels, and the results are shown in Fig. 7. There were three main metabolic pathways related to cardiovascular and cerebrovascular disease that were enriched in the differential genes between the groups (P < 0.05): the PI3K-Akt signaling pathway (ko04151), MAPK signaling pathway (ko04010), and Hippo signaling pathway (ko04390).

Genes related to cardiovascular and cerebrovascular diseases annotated by the PI3K-Akt signaling pathway included Fgf12, Fgfr1, PDGFA, and PDGFRα. Nfatc1, PDGFA, PDGFRα, Tgfbr2, Fgf12, and Fgfr1 were annotated by the MAPK signaling pathway. The genes related to cardiovascular and cerebrovascular disease annotated by the Hippo signaling pathway were Bmp4, Tcf7l2, and Wnt16. We found that PDGFA and PDGFRα genes were annotated both by the PI3K-Akt signaling pathway and MAPK signaling pathway, and their differentially methylated regions were located in the promoter region. Therefore, PDGFA and PDGFRα are considered to be target genes of high salt-induced hypertensive vascular endothelial lesions and salt memory. Subsequent studies will start with the PDGFA and PDGFRα genes for physiological experiment verification.

## Discussion

Studies have shown that blood stasis is positively correlated with the risk and severity of hypertension. Blood stasis is a major syndrome factor in the development of hypertension. When the body has excessive salt intake compared to the capacity of the kidney, "urinary sodium excretion dysfunction" can appear, which can significantly increase the extracellular fluid volume and cause an abnormal increase in the left ventricular filling pressure. This leads to increased cardiac output and renal afferent and efferent arteriole vascular resistance and causes blood pressure to rise^22^. Long-term high salt intake will lead to the synthesis and release of excessive eosinophils, inhibit the sodium pump, and thus increase the intracellular Na^+^ content. Moreover, the increased exchange of sodium and calcium will lead to calcium overload, which will cause the contraction of intravascular smooth muscle, increase the resistance of perivascular smooth muscle, and increase blood pressure^17^.

The present study demonstrates that salt-mediated inflammatory memory exists in the liver and contributes to the persistence of cardiovascular damage caused by high salt intake, in which the continuous repression of SIRT3 plays a critical role^12^. A reduction in salt intake has been widely accepted to lower blood pressure in hypertensive individuals, which contributes to reduced cardiovascular events^23^. The traditional view is that salt directly increases sodium and water retention in the body and activates sympathetic activity and the RAAS, thereby leading to cardiovascular dysfunction^12^. Previous studies have shown that high salt intake can cause vascular disease, which can lead to hypertension and other cardiovascular dysfunctions^24^. The findings of this investigation suggest that even after the establishment of hypertension and vascular damage, a reduction in salt intake does not reverse the vasculopathy that has already occurred. This observation raises the possibility that high salt-induced memory may contribute to the continuous pathology of the blood vessels.

ET-1 and NO are vasomotor factors secreted by VECs, which are important factors that reduce the stability of the vascular environment. Studies have found that when the body is in a state of hypertension vascular endothelial disease, a variety of vasoactive substances are destroyed, causing vasomotor imbalance and accelerating the development of hypertension. Additionally, the change in NO levels will cause an oxidative stress response and activate the NF-κB pathway. This will cause an inflammatory response in the body, promote the release of proinflammatory mediators in the vascular wall, and accelerate the occurrence and development of hypertension and its vascular complications^25^. ET can induce vasoconstriction of the most effective biological factor. It can also promote VSMC proliferation, fibrosis, and inflammatory reactions and is of great significance in the pathophysiology of vascular endothelium^26^. ET-1, a major member of the ET family, can activate vascular smooth muscle voltage-dependent Ca^2+^ channels, promote Ca^2+^ influx across the membrane, increase the intracellular free calcium concentration, activate the phosphatidylinositol-protein-activating enzyme system, and play an important role in the relaxation and contraction of blood vessels^27^. NO is produced intracellularly by l-arginine through NOS and is a diatomic free radical molecule. NO is known as the "guardian of the cardiovascular system". Studies have found that it can be used to dilate blood vessels, affect vascular growth, regulate blood pressure levels, and inhibit platelet adhesion and has a certain protective effect on vascular endothelial function^28^. In this study, the results showed that salt memory can continue to increase the expression levels of ET-1, which could be a critical reason for the continuous vascular endothelial dysfunction and high blood pressure.

TNF-α is a proinflammatory factor that is activated by activated monocytes and macrophages and appears at the very early stage of the inflammatory response. Some studies have shown that TNF-α has a direct damaging effect on VECs^29^. It is also one of the inflammatory mediators with the greatest impact on the body. After TNF-α binds to its receptor, it activates NF-κB, the MAPK signaling pathway and the JNK enzyme. It can promote the synthesis of other proinflammatory cytokines^30^ and regulate the inflammatory response of cells. TNF-α can accelerate the activation and aggregation of white blood cells. In such a state, the body will produce an inflammatory reaction, which increases the release of PDGFA, causes microcirculation disorder, increases the release of oxygen free radicals in the body, induces the release of the proinflammatory factor IL-6 from monocytes, damages VECs, promotes the proliferation of VSMCs, increases vascular resistance, and leads to an increase in blood pressure^31^. IL-1β is a multifunctional cytokine that is mainly activated by neurons, glial cells and VECs. It can activate a large number of downstream targets, promote blood coagulation in the body, promote VEC proliferation, and destroy the stability of atherosclerotic plaques. It mediates the process of impaired vascular endothelial contraction and relaxation^32^. In this study, we found that the levels of TNF-α and IL-1β showed an increasing trend in the HSD group and HSM group compared with the CON group, but this trend was not statistically significant.

Myocardial cells and endothelial cells are closely related in the coronary microvascular network. Endothelial cells are widely distributed on the surface of the heart cavity and are involved in hemostasis, vascular regulation, angiogenesis and other important processes. For patients with hypertension, poor blood pressure control can lead to vascular endothelial dysfunction, and the heart is one of the first organs to be damaged. Many studies have also found that various methylation-mediated pathways have a greater impact on myocardial cells. Based on this, we extracted heart tissue for DNA methylation sequencing. RRBS-heavy sulfite sequencing technology was selected for analysis. RRBS cuts DNA enzymes into small fragments, locates and selects genomic subunits for research, and conducts heavy sulfite sequencing analysis on important gene regulatory regions to evaluate the methylation level of CpG. According to the results, the PI3K-AKT signaling pathway, MAPK signaling pathway and Hippo signaling pathway may be the key pathways of high salt and salt memory-induced hypertensive vascular endothelial lesions, and PDGFA and PDGFRα are their target genes. The possible mechanism may be the inflammatory response, regulation of VSMCs, regulation of cardiac compensatory function, participation in oxidative stress, participation in autophagy, etc.

The PI3K/AKT signaling pathway plays an important role in regulating cell functions, such as cell differentiation, proliferation, transcription, angiogenesis, inflammatory response, energy metabolism, and protein synthesis^33,34^. AKT plays a major role in the physiological and pathological mechanisms of vascular remodeling, and AKT signaling protects endothelial function by reducing the proliferation of VSMCs and promoting their survival in vascular restenosis and atherosclerosis^35^. The PI3K/AKT/mTOR pathway is involved in the reverse regulation of autophagy. Downregulation of the activity of this pathway can reduce the inhibition of the regulatory release of autophagy initiation. Autophagy can inhibit the accumulation of inflammatory factors through degradation, resist oxidative stress damage, and protect the vascular endothelium^36^.

MAPK belongs to serine-threonine protein kinases, which are mainly involved in the ERK, p38MAPK and JNK pathways^37^. The MAPK signaling pathway is mainly involved in cell proliferation, differentiation and apoptosis, as well as the occurrence and development of inflammatory states in the body, and plays an important role in cytoskeletal recombination and transcriptional regulation^38^. When TNF-α levels increase, serum cells are stimulated by inflammatory factors, and MAPK kinase is phosphorylated successively. When the MAPK pathway is activated, cell functional activities are affected, and the reverse transcription factor NF-κB pathway is activated. The NF-κB pathway can regulate cell survival and apoptosis and the immune response of the body to a certain extent. It can also regulate the secretion of VCAM-1 and ICAM-1 by vascular endothelial cells, accelerate the adhesion of white blood cells to VSMCs, and aggravate the inflammatory response^39^.

The Hippo signaling pathway is involved in regulating cell functional activities in the body and has a great impact on the physiological and pathological processes of cardiovascular diseases. Thus, it has great potential in the treatment of cardiovascular and cerebrovascular diseases and in promoting changes in the organ state^40^. Studies have shown that the Hippo/TAZ pathway is involved in the occurrence and development of vascular structural and functional damage in hypertension and mediates vascular inflammation caused by hypertension^41^.

Platelet-derived growth factor (PDGF) can cause vasoconstriction, and its effect is stronger than that of angiotensin II. PDGF is a proinflammatory factor that can cause an inflammatory response in the body, promote the proliferation of endothelial cells, smooth muscle cells and fiber cells, and lead to the formation of new intima, whose continuous proliferation narrows the vascular cavity, causing thrombosis, infarction and other pathological phenomena^42^. PDGFA is the main mitogen of many cell types and belongs to the PDGF family^43^. PDGFA has two different tyrosine kinase receptors, PDGFRα and PDGFRβ, and its binding to these two receptors exerts different biological effects. As a specific receptor of PDGFA, PDGFRα is involved in a variety of pathways related to cardiovascular and cerebrovascular diseases and is of great significance for angiogenesis, inflammatory response, cell survival and apoptosis, cell proliferation and migration^44,45^. Studies have found a positive correlation between high PDGFRα expression and vascular invasion^43^. The Wnt signaling pathway is widely found in multicellular eukaryotes and can affect cell proliferation and migration and maintain cell activity. In the process of angiogenesis, the interaction between the Wnt signaling pathway and PDGFA is of great significance. It is beneficial for promoting angiogenesis^46^. After being stimulated by PDGF, the cells showed a spindle-shaped slender arrangement, and the stress fibers in the cells appeared and were densely arranged. Additionally, VSMC platy podia increased significantly^43^.

In this study, we reduced the concentration of salt solution from 12 to 4% and demonstrated the existence of a salt memory effect. However, 4% is still a considerably high concentration. In future work, we will observe this salt memory effect on blood pressure and vessels at different concentrations and may use customized feed with different salt concentrations that are closer to our diet. Additionally, increasing the sample size could strengthen the conclusions and provide more evidence of a high salt memory effect, which supports a salt-restricted diet. Due to funding limitations, we only selected a minimum sample size for the analysis of DNA methylation-related assays, which may introduce bias due to individual differences. Increasing the sample size in future studies can better minimize this type of bias. Furthermore, the current study did not consider the impact of feeding rhythm, and accordingly, we plan to include additional groups in our future research to observe and analyze this factor comprehensively, with the aim of gaining a more comprehensive understanding of the effects and mechanisms of salt memory.

## Conclusion

In conclusion, excessive salt intake may cause hypertension and vasculopathy, and our findings suggest that after the establishment of hypertension and vascular damage, a reduction in salt intake does not reverse the vasculopathy that has already occurred. This observation raises the possibility that high salt-induced memory may contribute to the continuous pathology of the blood vessels. Therefore, a salt memory phenomenon exists, and this memory effect may be correlated with the levels of DNA methylation. The PI3K-AKT signaling pathway, MAPK signaling pathway and Hippo signaling pathway may be the key pathways of high salt and salt memory-induced hypertensive vascular endothelial lesions, and PDGFA and PDGFRα are their target genes.

## Data Availability

The datasets generated and analysed during the current study are available on GenBank (SUB12918236) https://www.ncbi.nlm.nih.gov/sra/PRJNA943429. The BioProject ID is PRJNA943429, and will be made public upon acceptance. The original contributions present in the study are included in the article. Further inquiries can be directed to the corresponding author.
